# Factors related to compliance with periodontal disease treatment appointments: A literature review

**DOI:** 10.4317/jced.59752

**Published:** 2022-11-01

**Authors:** Rubiel A. Marín-Jaramillo, Andrés A. Agudelo-Suárez

**Affiliations:** 1 Periodontist. PhDc Epidemiology and biostatistics- Faculty of dentistry Universidad CES; 2 PhD Public health- Faculty of dentistry Universidad de Antioquia

## Abstract

Periodontal disease is considered a public health problem due to its high prevalence worldwide. Currently, the treatment of periodontal disease is not always successful due to many aspects, including non-compliance with scheduled appointments by the patient. Several authors over the years have evaluated different factors that could be related to non-compliance with periodontal treatment, so the objective of this narrative review of the literature was to explore and analyze the factors that may be related to compliance with periodontal treatment appointments. The results obtained allowed us to conclude that compliance by the patient will depend on a series of multiple factors, however, such compliance will favor the outcome of the periodontal treatment.

** Key words:**Patient Compliance, Treatment Adherence, Periodontal Diseases, (MeSH).

## Introduction

Periodontal disease is the result of an inflammatory process, usually chronic, that affects the dental supporting tissues. Periodontal diseases are divided into gingivitis and periodontitis. Periodontitis is an inflammation of the gingiva, characterized by bleeding on probing and loss of periodontal bone support. This inflammatory process is produced by the presence of polymicrobial biofilms inside the gingival sulcus. The presence of these biofilms is necessary, but not sufficient for the development of the disease (1). Different factors that may be related to the success or failure of treatments for this condition have been evaluated, including systemic conditions such as diabetes, cardiovascular and chronic inflammatory diseases; behavioral conditions such as smoking, use of illicit drugs and alcohol; and sociodemographic conditions such as educational and socioeconomic level, among others (2-4).

Within the behavioral aspects, it has been determined that compliance with periodontal therapy and maintenance appointments (also called “adherence” in the literature) could positively or negatively affect the onset or progression of periodontal disease. Adherence is defined by the WHO as “the extent to which a person’s behavior corresponds with agreed recommendations from a health care provider” and includes the commitment of the patient to the illness, its treatment, and the therapist (5). To improve adherence, patients must acquire an active role in the management of the disease, increase their autonomy and capacity for personal care, must know and understand the disease, its treatment, and the importance of its compliance, additionally, patients must have good communication with the professional, which allows them to actively participate in decision-making (6). Persistence, on the other hand, is related to the amount of time for which the patient continues the prescribed treatment, that is, the time from implementation to discontinuation. In this sense, for patients to be adherent, they must comply, and they must also be persistent. For the periodontal patient, persistence is a major factor because, as with any other chronic disease, treatment is for life, however, both adherence and persistence are complex concepts that go beyond yes or no (6). This narrative review of the literature aims to evaluate the concept of compliance, taking into account adherence, persistence, and other determining factors for its success.

Patient compliance with maintenance therapy is influenced by several factors, such as an excellent dentist-patient relationship, good communication, and the patient’s understanding of their oral health status. In this sense, compliance will depend on both, the professional and the patient, and will be a fundamental factor in the development and progression of periodontal disease. Most studies used for this review were conducted on patients going through the periodontal maintenance phase, due to the ease and relevance of evaluation. Periodontal Maintenance (PM) is defined by the American Academy of Periodontology as “Procedures performed at selected intervals to assist the periodontal patient in maintaining oral health.” This therapy includes updating medical and dental records, radiographic follow-up, soft tissue examination, periodontal evaluation, and removal and control of dentobacterial biofilm from crevicular and pocket areas by scaling and root planing when necessary (7,8). The objective of this narrative review of the literature was to analyze the factors related to patient compliance with periodontal maintenance appointments.

## Importance of compliance with periodontal treatment: A historical approach

The preservation of periodontal health of patients who received surgical or non-surgical treatment requires a strict and constant program. Transferring patients from active treatment to a maintenance program is an important step that requires time and effort, patients must understand the purpose of the maintenance program, as well as understand that periodontal disease is not cured, but controlled. Different studies developed by relevant authors in the history of periodontics have highlighted the importance of maintenance therapy. For example, Nyman, in 1977, evaluated the effects of maintenance after different surgical procedures in 25 patients. The procedures included apically repositioned flap surgery, with the elimination of bony defects; the Widman flap surgical technique with curettage of bony defects without removal of bone; the Widman flap technique with curettage of bone defects, without bone removal; and gingivectomy with curettage of bony defects without removal of bone. Patients who did not undergo maintenance therapy showed reappearance and increase in the accumulation of biofilm in the operated areas, leading to recurrence of the disease with significant further loss of clinical attachment level (9).

On the other hand, Axelsson and Lindhe, in 1981, carried out a clinical study with 90 patients to evaluate the effect of negligence in the provision of adequate maintenance care after periodontal treatment in a 6-year follow-up period. After presurgical root instrumentation and instruction in oral hygiene practices, all patients underwent modified Widman flap surgery. Then, one out of three patients stopped receiving treatment, while the other two entered a maintenance program once every 3 months. These patients maintained excellent oral hygiene and evidenced a very low frequency of bleeding. Additionally, probing depths and attachment levels remained unchanged over the 6 years. In contrast, patients who were not linked to maintenance therapy showed signs of recurrent periodontitis at 3 and 6 years (10).

Other findings were reported by Lindhe and Nyman, in 1984, from 75 patients with advanced periodontitis who had been successfully treated with non-surgical therapy and modified Widman flap surgery. After active therapy, the patients were subjected to maintenance appointments every 3 or 6 months. The authors reported that with adequate maintenance therapy, recurrence of the disease occurred in very few cases after 14 years. However, it should be noted that recurrent periodontitis appeared at unpredictable time intervals, but was concentrated in approximately 25% of the studied population (15 of 61). The authors suggested that, in a population highly susceptible to periodontitis, most patients can be controlled by providing optimal supportive periodontal therapy, while a relatively smaller proportion of patients (20-25%) will experience occasional episodes of recurrent periodontitis (11). A more recent study on long-term maintenance (10-year follow-up) of periodontal patients reported that joining a periodontal maintenance program, even when complex prosthetic procedures have been performed, guarantees a greater probability of achieving stability of periodontal health (12).

A longitudinal study evaluated the maintenance phase of periodontal patients for 9 to 11 years. The results showed that regular maintenance after treatment is associated with low levels of tooth loss. Regarding the interval between periodontal maintenance appointments, the literature shows a wide range of periods, including 2 weeks, 2-3 months, 3 months, 3-4 months, 3-6 months, and even 18 months. It was found in a systematic review that patients without a history of periodontal disease differ from periodontal patients, who require more meticulous therapy and less periodicity between maintenance appointments (7). However, there are no precise data on the optimal frequency of maintenance therapy that allows adequate control of the disease and prevents progression and future tooth loss (6,7,13).

## Factors that may affect compliance with periodontal treatment

Some authors have evaluated possible factors that could affect patient compliance with periodontal treatment appointments. Wilson *et al*. (1971) and Becker *et al*. (1984) reported the first studies on compliance with periodontal appointments, indicating that more than 34% of the patients did not return to the dental office and 50% were deficient in compliance. Likewise, patients who were not compliant presented less favorable periodontal health (14,15). From that point on, the evidence in the scientific literature has been consistent in promoting patient adherence to treatments to obtain the best possible long-term result (10,16,17).

A recent narrative review proposes that factors related to noncompliance could be categorized into different groups: those associated with the patient, the ones associated with the professional intervention, some psychological factors that include aspects related to behavior, and finally, those associated with the intervals between appointments (6). A retrospective study of 327 patients in the periodontal maintenance phase, evaluated the relationship between treatment compliance and average clinical probing depth, finding that less compliant patients had a statistically significant association with increased probing depth. This study also reported that individuals with the highest scores on a periodontal risk assessment were the least compliant (18).

According to the findings in this review, Table 1 shows the factors that could be related to compliance with periodontal disease treatment appointments, proposing a classification into two groups according to their nature: individual and contextual. Five contextual and nine individual factors were identified.

**Table 1:**
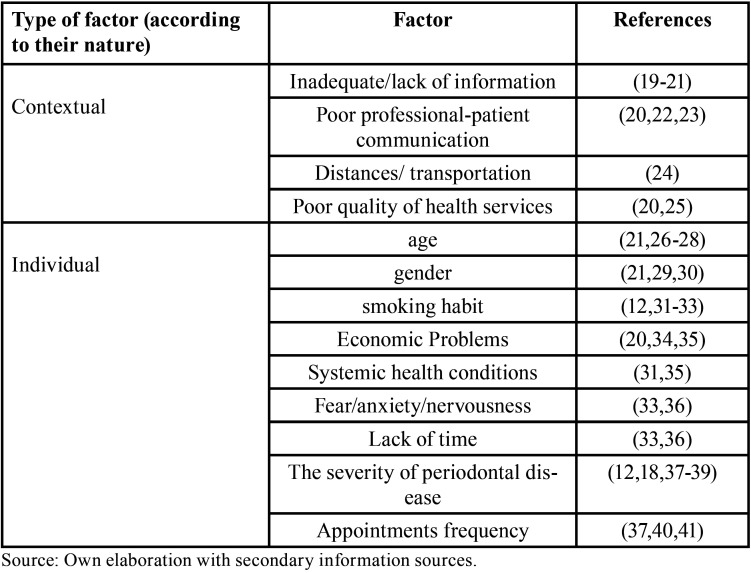
Factors related to compliance with periodontal treatment appointments.

Among the possible factors related to compliance with periodontal treatment appointments, poor communication between the professional and the patient (20,22,23), and age stand out, reporting that young people tend to be less compliant (21,26-28). Likewise, the literature reports that the severity of the disease is a factor closely related to compliance (12,18,37-39). Additionally, other contextual factors such as economics (20,34,35) and lack of time could also influence the commitment of the periodontal patient (33,36).

Gender was also reported as a possible influencing factor, where women seem to be more compliant, which has been attributed to a greater interest in appearance and health status in the female population. Similarly, the literature reports that the frequency between appointments is directly proportional to compliance, this may be associated with the patient’s commitment and understanding of their oral health. Finally, this review has made it possible to recognize the importance of the periodontist providing adequate information to patients from the beginning of the treatment to promote compliance (19-21).

**Figure 1:**
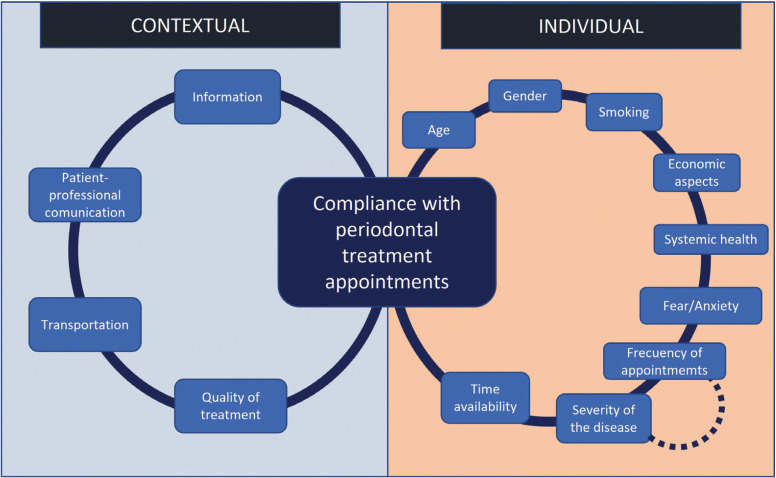
Factors related to compliance with periodontal maintenance appointments.

Figure 1 proposes four factors within the category called “contextual” (Lack of information, inadequate communication between professional and patient, transportation, and poor quality of treatment). These factors, which are external to periodontal patients, could negatively affect appointment compliance and could be modifiable with educational and government interventions. On the other hand, nine factors are identified in the category “Individual” (Gender, smoking habit, economic aspects, systemic health, fear/anxiety, lack of time, frequency of appointments, and severity of the disease). Of which, the frequency between maintenance appointments is usually of relevant importance since the more times per year the patient must attend, the more likely that contextual and individual factors may affect compliance. The frequency between appointments is variable and is determined by the individual risk assessment of each patient, but the authors agree that periods of three months are ideal for the success of periodontal maintenance.

Future perspectives on compliance with periodontal treatment appointments.

The study of the compliance of periodontal patients with the appointments required for their treatment is complex. Intervention strategies to promote compliance in this population often fall short of clinician ideals. At present, the study plans of the different periodontal programs around the world do not usually include communication strategies for specialists in training, which represents a problem when it is visualized that, in the first meeting between the patient and the professional, it is possible to generate a bond that motivates the patient to commit to the proposed treatment.

According to The Global Burden of Disease (GBD), periodontitis is the sixth most prevalent disease in the world (42), and due to its high prevalence, it is considered a public health problem. In the short and medium-term, periodontal research should focus on the development of strategies that favor the success of long-term periodontal treatment by strengthening the compliance of periodontal patients. Evidence reports that motivational interviewing could facilitate long-term patient management (43,44). On the other hand, current research should focus on the identification of predictors of compliance, including those individual and contextual factors commonly overshadowed by periodontal clinical parameters but which are of great relevance in the pathophysiological process of the disease, as well as in the design and the execution of mixed (quantitative and qualitative) and longitudinal studies, which allow a better understanding of the real behavior of the disease at a social environment. In this way, intervention strategies focused on minimizing the negative impact of tooth loss due to periodontal disease could be developed.

## Conclusions

Compliance with periodontal maintenance appointments favors the reduction of biofilm and gingival bleeding rates, thus reducing the probability of disease progression and, ultimately, tooth loss. Although compliance is a difficult phenomenon to assess since it is the result of interactions of multiple factors, it must be recognized as one of the main aspects that could predict the success of periodontal treatment. New studies are needed to evaluate this predictive capacity and promote the development of public health strategies focused on the elimination of contextual barriers that could hinder compliance with periodontal treatment.
